# Supplementary material for: Establishing the Clinical Utility of ctDNA Analysis for Diagnosis, Prognosis, and Treatment Monitoring of Retinoblastoma: The Aqueous Humor Liquid Biopsy
Source: Cancers (Basel). 2021 Mar 13;13(6):1282. doi: 10.3390/cancers13061282 (PMC8001323; doi:10.3390/cancers13061282)
Supplement: Supplementary file 1 [file cancers-13-01282-s001.pdf]

cancers MDPI

Supplemental Materials:

Table S1. REMARK Checklist.

	Item to be reported	Page no.
	**INTRODUCTION**	
1	State the marker examined, the study objectives, and any pre-specified hypotheses.	6
	**MATERIALS AND METHODS**	
	Patients	
2	Describe the characteristics (e.g., disease stage or co-morbidities) of the study patients, including their source and inclusion and exclusion criteria.	7–8
3	Describe treatments received and how chosen (e.g., randomized or rule-based).	Supplemental file 2
	Specimen characteristics	
4	Describe type of biological material used (including control samples) and methods of preservation and storage.	Supplemental file 2
	Assay methods	
5	Specify the assay method used and provide (or reference) a detailed protocol, including specific reagents or kits used, quality control procedures, reproducibility assessments, quantitation methods, and scoring and reporting protocols. Specify whether and how assays were performed blinded to the study endpoint.	Supplemental file 2
	Study design	
6	State the method of case selection, including whether prospective or retrospective and whether stratification or matching (e.g., by stage of disease or age) was used. Specify the time period from which cases were taken, the end of the follow-up period, and the median follow-up time.	7
7	Precisely define all clinical endpoints examined.	8
8	List all candidate variables initially examined or considered for inclusion in models.	Supplemental file 2
9	Give rationale for sample size; if the study was designed to detect a specified effect size, give the target power and effect size.	7
	Statistical analysis methods	
10	Specify all statistical methods, including details of any variable selection procedures and other model-building issues, how model assumptions were verified, and how missing data were handled.	Supplemental file 2
11	Clarify how marker values were handled in the analyses; if relevant, describe methods used for cutpoint determination.	Supplemental file 2
	**RESULTS**	
	Data	
12	Describe the flow of patients through the study, including the number of patients included in each stage of the analysis (a diagram may be helpful) and reasons for dropout. Specifically, both overall and for each subgroup extensively examined report the numbers of patients and the number of events.	8
13	Report distributions of basic demographic characteristics (at least age and sex), standard (disease-specific) prognostic variables, and tumor marker, including numbers of missing values.	Table 1
	Analysis and presentation	
14	Show the relation of the marker to standard prognostic variables.	Previously published in citation 14
15	Present univariable analyses showing the relation between the marker and outcome, with the estimated effect (e.g., hazard ratio and survival probability). Preferably provide similar analyses for all other variables being analyzed. For the effect of a tumor marker on a time-to-event outcome, a Kaplan-Meier plot is recommended.	11
16	For key multivariable analyses, report estimated effects (e.g., hazard ratio) with confidence intervals for the marker and, at least for the final model, all other variables in the model.	Previously published in citation 14
17	Among reported results, provide estimated effects with confidence intervals from an analysis in which the marker and standard prognostic variables are included, regardless of their statistical significance.	Previously published in citation 14
18	If done, report results of further investigations, such as checking assumptions, sensitivity analyses, and internal validation.	11

	**DISCUSSION**	
19	Interpret the results in the context of the pre-specified hypotheses and other relevant studies; include a discussion of limitations of the study.	11
20	Discuss implications for future research and clinical value.	13-14

Methods S1. Detailed Methods Section

Specimen Collection and Storage

As previously described [1,2], each liquid biopsy sample consisted of up to 100ul of AH taken from the anterior chamber via clear corneal paracentesis with a 32-gauge needle [3]. Eyes with any degree of anterior segment involvement were not sampled. This included eyes that had intraocular pressure >22mm Hg, a shallow anterior chamber, or a compromised view of the anterior chamber structures for any reason. Throughout the procedure, the needle remained bevel-up over the pharmacologically dilated iris and did not contact the iris, lens, vitreous cavity, or tumor. The anterior chamber shallowed slightly but remained formed during paracentesis, and the needle site was examined for any leakage after AH sampling. Immediately following specimen collection, AH samples were handed off directly in the operating room and placed on dry ice. They were then stored at −80 ℃ and underwent cfDNA isolation within 72 hours of extraction using the QIAamp Circulating Nucleic Acid Kit (Qiagen) [1,2]. The concentration of cfDNA in the AH was measured via Qubit high sensitivity dsDNA assay.

Matched blood samples were also collected from patients at diagnosis via venipuncture and were stored at room temperature in disodium EDTA collection tubes (Becton Dickinson Franklin Lakes, NJ, USA). Processing of blood samples was completed within 72 hours as previously described [4]. If enucleation was required during the course of treatment, tumor tissue was subsequently obtained either directly by the surgeon, post-enucleation, or from formalin-fixed and paraffin-embedded blocks for further genetic analyses [5].

Genomic Analysis of AH Samples

Genomic analysis of AH cfDNA was previously described in depth and based on established methods of somatic copy number alteration (SCNA) analysis [1,2,6,7]. Briefly, cfDNA was constructed into whole genome libraries that underwent shallow sequencing at 0.3× for copy number profiling. SCNAs were considered to be present at 20% deflection from a baseline human genome [2,6,7]. Genomic analyses were similarly performed on blood and tumor samples (when available).

Determination of cfDNA Tumor Fraction

The fraction of tumor DNA (TFx) for each sequenced AH cfDNA sample was calculated using ichorCNA software, available at https://github.com/broadinstitute/ichorCNA. Its algorithm of using a hidden Markov model to predict large-scale SCNAs within sequenced cfDNA has been described in detail for liquid biopsy platforms [5,8,9].

RB1 Variant Analysis of AH Samples

For detection of *RB1* pathogenic variants, the same whole genome libraries were further amplified to ~ 500 ng with 7–10 cycles of Polymerase Chain Reaction (PCR) for capture-based targeted next-generation sequencing (NGS) as previously described [5]. Bioinformatics analysis was performed to detect and to characterize single-nucleotide variant (SNV) using an in-house pipeline based on the bcbio pipeline at the CHLA Center for Personalized Medicine [5]. The presence of somatic variants in the AH, and tumor when available, were called as the consensus of VarDict and Mutext2 compared to the paired normal blood sample. Variant annotation was conducted with Ensembl VEP (v96).

Clinical Evaluation

All patients with RB were examined under anesthesia (EUA) for complete staging prior to AH sampling in order to assess extent and severity of disease. Tumors were classified based on International Intraocular Retinoblastoma Classification (IIRC) guidelines and TNM staging for RB [10,11]. After AH sampling (described below), treatment was non-randomized per CHLA protocol [12,13]. The treating physicians were blinded to the results of the AH analysis, and all treatment decisions were made per routine standard of care. All genetic and genomic findings remained separate from clinical data until final analysis. All participants have at least 12 months of follow-up after diagnosis in order to monitor the status of the intraocular tumor as per routine, as well as monitoring for adverse effects at the paracentesis site and extraocular disease.

Participants' charts were reviewed for clinical features including gender, age at diagnosis, laterality, tumor classification, seeding morphology, specific treatments used, and duration of follow-up (including EUA and in clinic).

Table S2. Single Nucleotide Variant Mutation Details.

Case_ID	SNV			
	RB1 gene SNV	%VAF	(unaltered, altered)	95% CI
33	ND	ND	ND	ND
44_OD	c.1666C>T, p.Arg556*	66.67%	37,74	(57.09–75.33%)
44_OS	c.1666C>T, p.Arg556*	100.00%	0,351	(98.95–100%) *
45	c.1422-1G >A, splice_acceptor_variant	96.60%	12,341	(94.7–98.5%)
46	c.1363C>T, p.Arg455*	99.15%	3,469	(98.6–100%)
47	c.958C>T, p.Arg320*	87.01%	10,68	(79.7–94.6%)
48	ND	ND	ND	ND

NA: not available; ND: not detected; * one-sided, 97.5%CI

Reference

1. Berry, J.L.; Xu, L.; Murphree, A.L.; Krishnan, S.; Stachelek, K.; Zolfaghari, E.; McGovern, K.; Lee, T.C.; Carlsson, A.; Kuhn, P.; et al. Potential of Aqueous Humor as a Surrogate Tumor Biopsy for Retinoblastoma. *JAMA Ophthalmology* **2017**, *135*, 1221–1230. doi:10.1001/jamaophthalmol.2017.4097
2. Berry, J.L.; Xu, L.; Kooi, I.; Murphree, A.L.; Prabakar, R.K.; Reid, M.; Stachelek, K.; Le, B.H.A.; Welter, L.; Reiser, B.J.; et al. Genomic cfDNA Analysis of Aqueous Humor in Retinoblastoma Predicts Eye Salvage: The Surrogate Tumor Biopsy for Retinoblastoma. *Mol. Cancer Res.* **2018**, *16*, 1701–1712. doi:10.1158/1541-7786.MCR-18-0369
3. Munier, F.L.; Soliman, S.; Moulin, A.P.; Gaillard, M.C.; Balmer, A.; Beck-Popovic, M. Profiling safety of intravitreal injections for retinoblastoma using an anti-reflux procedure and sterilisation of the needle track. *Br J. Ophthalmol.* **2012**, *96*, 1084-1087. doi:10.1136/bjophthalmol-2011-301016
4. Berry, J.L.; Xu, L.; Polski, A.; Jubran, R.; Kuhn, P.; Kim, J.W.; Hicks, J. Aqueous Humor Is Superior to Blood as a Liquid Biopsy for Retinoblastoma. *Ophthalmology* **2020**, *127*, 552–554. doi:10.1016/j.ophtha.2019.10.026
5. Xu, L.; Shen, L.; Polski, A.; Prabakar, R.K.; Shah, R.; Jubran, R.; Kim, J.W.; Biegel, J.; Kuhn, P.; Cobrinik, D.; et al. Simultaneous identification of clinically relevant RB1 mutations and copy number alterations in aqueous humor of retinoblastoma eyes. *Ophthalmic. Genet* **2020**, 10.1080/13816810.2020.1799417, 1–7. doi:10.1080/13816810.2020.1799417
6. Baslan, T.; Kendall, J.; Rodgers, L.; Cox, H.; Riggs, M.; Stepansky, A.; Troge, J.; Ravi, K.; Esposito, D.; Lakshmi, B.; et al. Genome-wide copy number analysis of single cells. *Nat. Protoc.* **2012**, *7*, 1024–1041. doi:10.1038/nprot.2012.039
7. Baslan, T.; Kendall, J.; Rodgers, L.; Cox, H.; Riggs, M.; Stepansky, A.; Troge, J.; Ravi, K.; Esposito, D.; Lakshmi, B.; et al. Corrigendum: Genome-wide copy number analysis of single cells. *Nat. Protoc.* **2016**, *11*, 616. doi:10.1038/nprot0316.616b
8. Polski, A.; Xu, L.; Prabakar, R.; Kim, J.W.; Cobrinik, D.; Hicks, J.; Berry, J.L. Longitudinal aqueous humor sampling reflects treatment response in retinoblastoma patients. *Invest. Ophthalmol. Visual Sci.* **2020**, *61*, 1394–1394.
9. Adalsteinsson, V.A.; Ha, G.; Freeman, S.S.; Choudhury, A.D.; Stover, D.G.; Parsons, H.A.; Gydush, G.; Reed, S.C.; Rotem, D.; Rhoades, J. Scalable whole-exome sequencing of cell-free DNA reveals high concordance with metastatic tumors. *Nat. Comm.* **2017**, *8*, 1–13.
10. Linn Murphree, A. Intraocular retinoblastoma: the case for a new group classification. *Ophthalmol. Clin. North Am.* **2005**, *18*, 41–53, viii. doi:10.1016/j.ohc.2004.11.003

11. Mallipatna, A. et al. in AJCC Cancer Staging Manual Vol. 8th Edition (eds M. B. Amin, S. B. Edge, & F. L. Greene) Ch. 68, 819–831 (Springer, 2017).
12. Berry, J.L.; Shah, S.; Bechtold, M.; Zolfaghari, E.; Jubran, R.; Kim, J.W. Long-term outcomes of Group D retinoblastoma eyes during the intravitreal melphalan era. *Pediatr. Blood Cancer* **2017**, *64*. doi:10.1002/pbc.26696
13. Berry, J.L.; Jubran, R.; Kim, J.W.; Wong, K.; Bababeygy, S.R.; Almarzouki, H.; Lee, T.C.; Murphree, A.L. Long-term outcomes of Group D eyes in bilateral retinoblastoma patients treated with chemoreduction and low-dose IMRT salvage. *Pediatr. Blood Cancer* **2013**, *60*, 688–693. doi:10.1002/pbc.24303
